# Recent Progress of Black Silicon: From Fabrications to Applications

**DOI:** 10.3390/nano11010041

**Published:** 2020-12-26

**Authors:** Zheng Fan, Danfeng Cui, Zengxing Zhang, Zhou Zhao, Hongmei Chen, Yanyun Fan, Penglu Li, Zhidong Zhang, Chenyang Xue, Shubin Yan

**Affiliations:** 1 Key Laboratory of Instrumentation Science & Dynamic Measurement, Ministry of Education, North University of China, Taiyuan 030051, China; s1906119@st.nuc.edu.cn (Z.F.); cuidanfeng@nuc.edu.cn (D.C.); zengxingzhang88@163.com (Z.Z.); Zzzzhou95@163.com (Z.Z.); lmxchm@126.com (H.C.); 18103510614@163.com (Y.F.); lipenglu98@163.com (P.L.); zdzhang@nuc.edu.cn (Z.Z.); 2 The School of Electrical Engineering, Zhejiang University of Water Resources and Electric Power, Hangzhou 310018, China; 3 Zhejiang-Belarus Joint Laboratory of Intelligent Equipment and System for Water Conservancy and Hydropower Safety Monitoring, Zhejiang University of Water Resources and Electric Power, Hangzhou 310018, China

**Keywords:** black silicon, light absorption enhanced, micro-nano manufacturing, nanometer surface

## Abstract

Since black silicon was discovered by coincidence, the special material was explored for many amazing material characteristics in optical, surface topography, and so on. Because of the material property, black silicon is applied in many spheres of a photodetector, photovoltaic cell, photo-electrocatalysis, antibacterial surfaces, and sensors. With the development of fabrication technology, black silicon has expanded in more and more applications and has become a research hotspot. Herein, this review systematically summarizes the fabricating method of black silicon, including nanosecond or femtosecond laser irradiation, metal-assisted chemical etching (MACE), reactive ion etching (RIE), wet chemical etching, electrochemical method, and plasma immersion ion implantation (PIII) methods. In addition, this review focuses on the progress in multiple black silicon applications in the past 10 years. Finally, the prospect of black silicon fabricating and various applications are outlined.

## 1. Introduction

**Figure 1:**
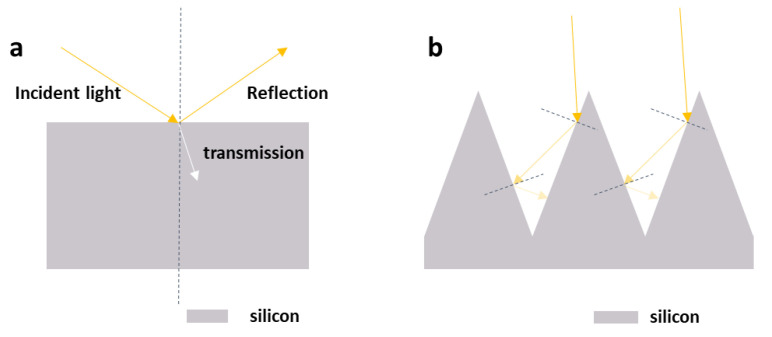
The reflection performance of (**a**) polished silicon and (**b**) black silicon.

Black silicon material is a silicon material with a micro-scale, nano-scale, and both micro-nano scales, including various structures such as holes, needles, and columns structures on the surface of wafers [1,2,3,4,5]. Compared with polished silicon, due to the micro-nano structure and impurity doping of the black silicon surface, it has ultralow light reflection in the visible and near-infrared bands, especially in the near-infrared band. Because of the limitation of the forbidden bandwidth of the polished silicon itself, when the wavelength is greater than 1100 nm [6], the light absorption ability is extremely reduced. The black silicon material has a light absorption rate of more than 90% in the range of 1100 nm to 2500 nm [7,8]. While extending the absorption range of materials in the longer band, it also improves the light absorption capacity. Black silicon has high absorption of light, as even the absorption rate is close to 100%. It is dark black to the naked eye. The micro-nano structure on the surface is the crucial factor to improving the absorption of this unusual material in the visible light band. Figure 1 explains the high absorption principle of the material. The incident light will be reflected multiple times between the tapered structures. Each reflection process is accompanied by transmission at the interface. Each transmission increases the number and probability of absorption of incident light by black silicon so that the light seems to be trapped in the micro-awl of the material surface. The existence of the light trapping effect is the main reason why black silicon has not only extreme low reflex in a broad spectral range, but also high absorption in the visible light band.

Since the discovery of black silicon materials in the late 20th century [9], after more than 20 years of development, black silicon has been fabricated in a variety of ways. Since the black silicon was discovered, common fabricating methods include nanosecond or femtosecond laser irradiation [8,9,10,11], metal-assisted chemical etching (MACE) [12,13,14,15], reactive ion etching (RIE) [16,17,18], wet chemical etching [19], and plasma immersion ion implantation (PIII) methods. Each fabrication method can fabricate black silicon with a micro-nano surface structure. However, the surface structure of the black silicon fabricated is also different using different fabrication processes. Furthermore, the material exhibits different optical characteristics and electrical characteristics. The choice of micro-nano processing technology also determines the performance of the device.

**Figure 2:**
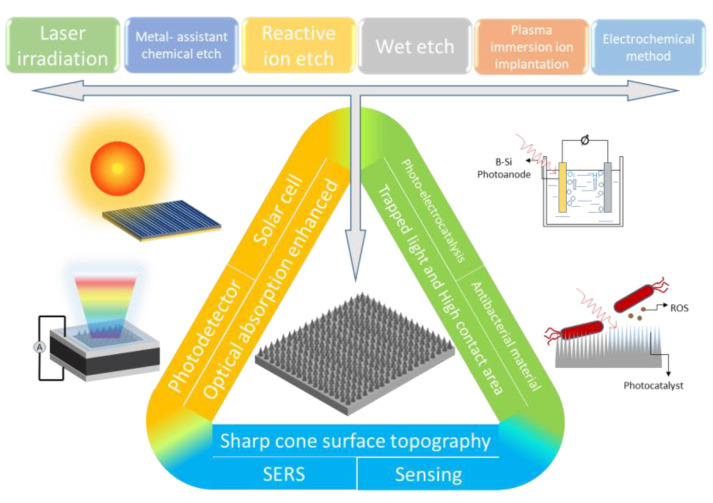
A figure overview of this review.

The development of black silicon materials has a lot of applications in different fields while the fabricating process is developing. In solar cells [20,21,22,23,24], the low reflectivity of black silicon and the high absorption of visible light is of great help to promote the performance of solar cells. In catalysis [25,26], the use of black silicon as a photoelectric catalytic substrate can synergize the light absorption characteristics of black silicon and the redox characteristics of photocatalysts to improve the performance of photocatalytic materials. In photodetectors [27,28,29,30,31], due to the excellent light absorption capabilities of black silicon in the visible and near-infrared bands, it can fabricate the silicon-based photodetectors with high responsivity and wide response ranges in the near-infrared band. In sensing applications, high-sensitivity detection can be achieved in surface-enhanced Raman scattering (SERS), biochemical sensing, gas sensing, etc. [32,33,34,35]. Figure 2 shows an overview of the entire review, including fabrication methods and characteristics and corresponding applications.

In this paper, we review the fabrication methods and applications of this special material over the last decade. The paper compares the complexity, cost, and suitable conditions of different fabricating processes. In addition, this paper also summarizes the researches on the optical properties of black silicon samples. Finally, we propose the outlook in fabrication methods and multiple applications.

## 2. Fabrication Techniques

### 2.1. Femtosecond/Nanosecond Laser Irradiation

**Figure 3:**
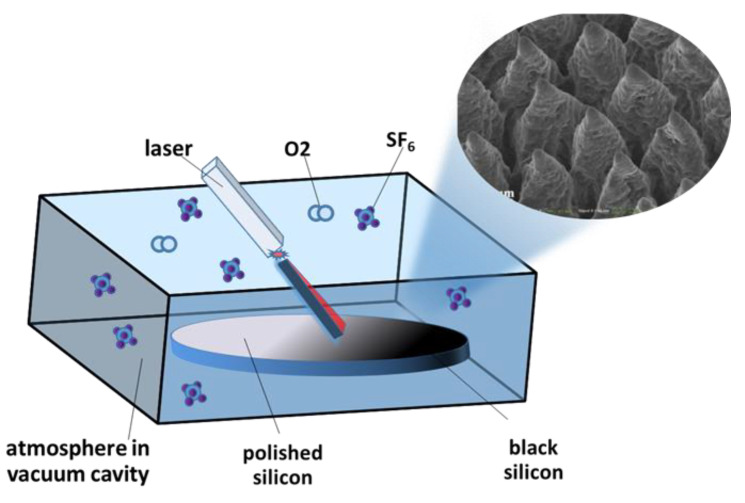
The fabrication principle of black silicon using femtosecond/nanosecond laser irradiation. Adapted from reference [37], with permission from Elsevier © 2020.

The fabrication of black silicon by laser irradiation is one of the most frequently used methods. According to different laser pulse lengths, the laser irradiation methods of preparing black silicon can be divided into femtosecond laser irradiation and nanosecond laser irradiation. Figure 3 shows the principle of femtosecond/nanosecond laser irradiation in the black silicon fabrication process [36]. In 1998, professor E. Mazur [9] and his team fabricated black silicon materials for the first time by using femtosecond lasers to irradiate the silicon surface under the corrosive gas SF_6_.

**Figure 4:**
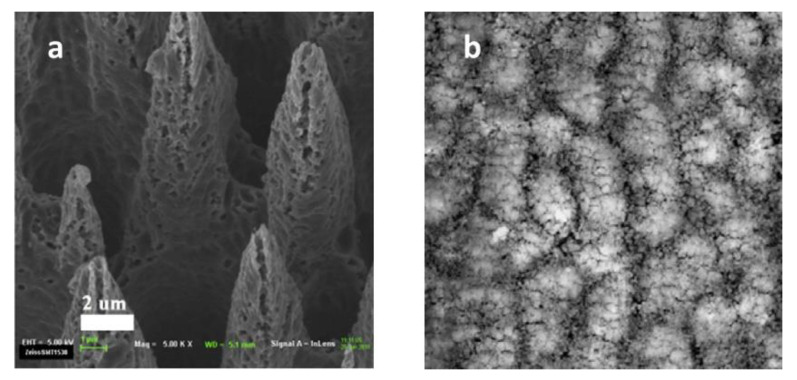
SEM image of black silicon fabricated by femtosecond laser irradiation in corrosive gas or air. (**a**) Corrosive gas atmosphere. Adapted from reference [37], with permission from Elsevier © 2020. (**b**) Air atmosphere. Adapted from reference [10], with permission from Elsevier © 2020.

In the last decade, the method of making black silicon by laser irradiation has been further developed. However, the atmosphere of the laser irradiation process is an extraordinarily important factor for the light absorption of black silicon, especially the absorption range. Due to the doping of element Sulphur, the light absorption range of black silicon was expanded to the near-infrared band in the Sulphur-bearing atmosphere. H. Mei [37] et al. developed a new type of black silicon with flexible properties by femtosecond laser irradiation. Figure 4a show the SEM image of black silicon using femtosecond laser irradiation in corrosive gas atmosphere. When manufacturing black silicon, the silicon wafer is placed on a programmable three-dimensional mobile platform, and the automatic irradiation can be realized through programming control. The surface nanostructures of black silicon are more regular, with an absorption rate of up to 97% in visible light, and they are insensitive to changes in incidence angle. Usually, the irradiation is done in the existence of corrosive gases to expand the light absorption range. However, A. Viorobyev [10] et al. fabricated black silicon by irradiating a high-intensity femtosecond laser directly on the silicon surface in an air atmosphere. Figure 4b show the SEM and absorption of black silicon using femtosecond laser irradiation in air atmosphere. The black silicon has low reflectance and an equidistant parallel nanostructured textured micro-slot array. The black silicon formed was a black velvet color, and the surface nanostructure was similar to the ridge shape when observed under SEM images. The reflectivity of black silicon under visible light is very good, only 3–4%.

In recent years, the effect of black silicon materials with different fabrication details on the absorptivity was also studied [38], such as the different doping elements, different irradiation power, and surface metal influence. In 2016, C. Li [39] et al. used phosphorus-doped silicon instead of sulfur-doped silicon to fabricate black silicon. Black silicon was fabricated by femtosecond laser irradiation under the SF_6_ atmosphere. After thermal annealing at 873 k in argon for 30 min, the black silicon still has a 70% absorptivity in the band from 1100 nm to 2000 nm. In 2017, X. Yu et al. [40] fabricated black silicon by femtosecond pulse laser irradiation on silicon wafers coated with gold film. The absorptivity of black silicon fabricated under different irradiation powers was researched. The results showed that the black silicon fabricated under different irradiation powers had no significant effect on the absorptivity. In a certain power range, the sample absorption rate increases with the increase of laser power. In 2018, Y. Su et al. [41] used a femtosecond laser to directly irradiate silicon wafers with the Al membrane on the surface to fabricate in the atmosphere. In the visible light region, the presence or absence of the Al membrane has little influence on the absorption rate, which is about 95%. However, at longer wavelengths, silicon wafers with an Al film have better absorptivity.

Black silicon is produced likewise by irradiating the surface of a silicon wafer with a nanosecond laser [42]. The fabricated black silicon sample also has very good optical and electrical properties. In 2015, B. Franta et al. [43] fabricated high-doped black silicon with high crystallization and high absorption by irradiating high-doped silicon wafers with nanosecond laser pulses. By comparing SEM images, the nanoscale surface of black silicon irradiated by a nanosecond laser is smoother than that irradiated by a femtosecond laser. The absorptivity of the black silicon sample is about 95% in the visible band. In 2018, L. Zhang et al. [44] fabricated black silicon by ablating silicon wafers with an infrared nanosecond pulse laser, and the researchers investigated the corresponding relation between the laser burning period and the surface morphology. The results showed that the black silicon microcolumn was formed by the accumulation of irradiation, instead of directly forming a columnar microstructure.

The material fabricated by laser irradiation can control the nanostructure on the surface to a certain extent by different time and laser power. More importantly, the laser irradiation method can dope the element to limit impurity level, which can improve adsorption in the near-infrared band. This method can fabricate high-performance black silicon to apply to wideband photodetectors. However, the femtosecond laser and nanosecond laser instruments are expensive and not suitable for manufacture and mass production.

### 2.2. Metal Assisted Chemical Etching

**Figure 5:**
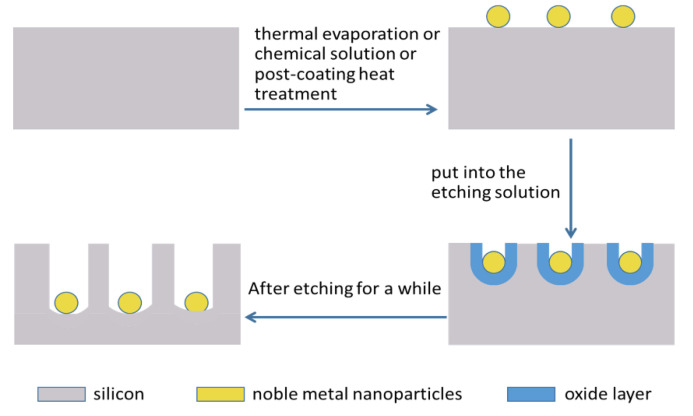
The fabrication principle of black silicon using metal assisted chemical etching (MACE).

The fabrication of black silicon by MACE has been common in recent years. The researchers fabricate black silicon by etching wafers with different metals, most commonly gold and silver. In general, metal nanoparticles are formed on the surface in different ways after the surface is cleaned and deoxidized. Then, the sample was put into the etching solution, and the nano-conical structure was formed on the material surface through the catalytic action of surface metal nanoparticles. The noble metal nanoparticles accomplish a vertical shield machine-like action. Figure 5 shows this activity, where black silicon formed in the MACE method [45]. Equations (1) and (2) explain the chemical process of the MACE method with gold nanoparticles as an example.

(1) $$H_{2}O_{2}+2H^{+}\overset{Au}{\to }2H_{2}O+2h^{+}$$

(2) $$Si+4HF_{2}^{-}+2h^{+}\to SiF_{6}^{2-}+2HF+H_{2}\left(g\right)$$

The main methods used to deposit metal nanoparticles are thermal evaporation, post-coating heat treatment, and chemical solution formation. Then, the MACE method is introduced in different deposition methods of noble metal nanoparticles.

**Figure 6:**
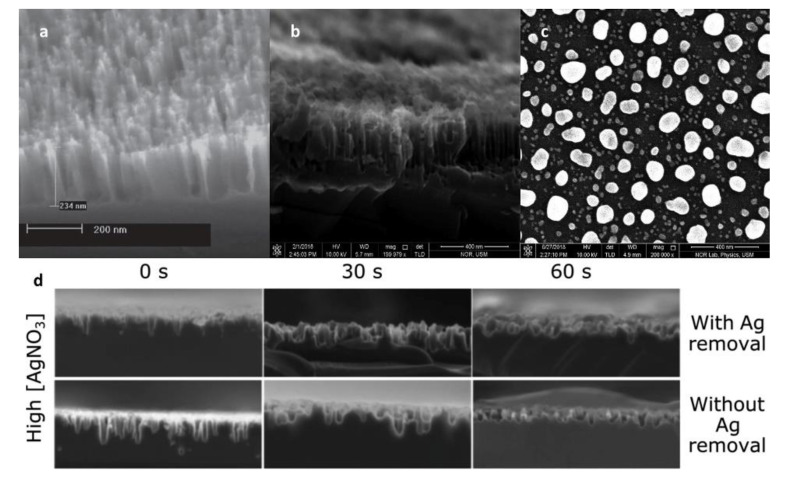
SEM of using different nanoparticles deposited methods to fabricate black silicon. (**a**) The thermal evaporation method using thermal evaporation instruments. Adapted from reference [24], with permission from John Wiley and Sons © 2020. (**b**,**c**) Using a post-coating heat treatment method to form noble metal nanoparticles. Adapted from reference [46], with permission from Elsevier © 2020. (**d**) Different etching time in high concentration Ag^+^ solution. Adapted from reference [53], with permission from IEEE © 2020.

The thermal evaporation method requires the use of thermal evaporation instruments to complete the evaporation of noble metal nanoparticles. Y. Liu et al. [24] fabricated black silicon by MACE. Surface noble metal nanoparticles are first formed by thermal evaporation. After the sample was immersed in the etching solution for a few minutes, a nano-tapered structure is created on the silicon chip. Figure 6a shows the SEM of the thermal evaporation method using thermal evaporation instruments. The reflectivity of the fabricated black silicon ranges from 300 nm to 1000 nm and is about 8%.

In 2019, N. Noor et al. [46] used a post-coating heat treatment method to form noble metal nanoparticles. Silver assisted etching was used as the fabrication method. Researchers studied the influence of annealing temperature on the surface morphology and optical properties of the material. After removing the natural oxide from the monocrystalline silicon sheet, 15 nm Ag film was formed on the silicon wafer using radio frequency sputtering. Figure 6b,c show the cross-section and top-view SEM of black silicon using a post-coating heat treatment method. The silver nanoparticles were formed by hot annealing at 200 °C, 230 °C, and 260 °C for 40 min in a nitrogen environment. Finally, black silicon was prepared by placing the sample in an etching solution. The reflectance of black silicon was only 3% at the 600 nm wavelength of the sample annealed at 230 °C.

The formation of noble metal nanoparticles from chemical solutions is one of the most common and low-cost methods. To fabricate black silicon materials using this method, the cleaned silicon wafers are first put into a mixture of noble metallic ions to model noble metal nanoparticles on the surface. After that, the sample was placed into the etching solution for a while, and the nanostructure was formed on the surface through the catalysis of nanoparticles. Figure 6d shows SEM of black silicon fabricated by the MACE method in different etching time in high concentration Ag^+^ solution.

Y. Su et al. [47] fabricated black silicon by MACE. The researchers first used alkaline etching to form micro columnar structures on the surface of silicon wafers. The sample was immersed in etching mixed solution for 8 min at no heating situation. After gold nanoparticles were formed, the solution was further etched to form a microporous structure on the surface. At 200 nm to 1000 nm, the absorption rate was about 85%. In 2014, C. Wang et al. [48] also fabricated double-layered black silicon by alkaline anisotropic etching and MACE. In 2014, Z. Zhao et al. [49] fabricated black silicon by MACE. It was also the first to use an alkaline etching method to form a micro pyramid morphology on the surface, and then to form micro holes in the micro pyramid structure by etching solution with silver ions. In the presence of an antireflection layer, black silicon has a reflectivity of less than 5% in the 400 nm to 1000 nm band. In 2016, H. Zhong et al. [50] fabricated nanostructured black silicon by MACE. In the production, the alkaline etching is eliminated, and the MACE is directly used. The nanostructure was etched on the material, and the absorptivity reached 95% at the band of 400 nm to 1100 nm. In 2019, I. Putra et al. [51] used alkaline etching to form tiny pyramidal structures on the surface. Micro-pores were formed in the micro-pyramid structure by the silver ion etching solution. The reflectance of the sample was less than 5% in the band of 300 nm to 900 nm. In 2019, F. Hu et al. [52] fabricated black silicon by MACE. First, the silver is formed on the surface of the silicon by heating it under low pressure. Then, the black silicon was immersed in a mixture etching solution at room temperature. Under the catalysis of silver, a micro-conical surface structure is formed. The absorptivity of black silicon with silver film at 400 nm to 1000 nm is more than 75%. The absorptivity of black silicon with Ag-NPs from 400 nm to 1000 nm is about 90%. Due to the non-impurity-doped, the absorptivity decreases seriously at the wavelength of 1000 nm to 2200 nm.

The researchers not only used MACE to fabricate black silicon, but also explored the optimization of the preparation process [46] through experiments. When a different etching time [54,55], solution metal ion density [53], or substrate thickness are used, the fabricated black silicon sample will have different optical properties.

In 2009, H. Branz et al. [56] fabricated black silicon materials by mixing 5 nm colloidal gold particles or HAuCl_4_ solution in the etching solution. The results showed that the black silicon made of colloidal gold particles mixed with 5 nm solution had better anti-reflection ability, and the reflectivity was less than 8% between 300 nm and 1000 nm. Moreover, the formation mechanism of nanopores on the black silicon surface is catalyzed by Au. The catalytic decomposition of hydrogen peroxide on the gold nanoparticles leads to rapid local oxidation of Si. Then hydrofluoric acid etches SiO_2_ to form it. In 2015, S. Marthi et al. [57] fabricated black silicon by MACE, and the researchers studied the effects of metal-assisted chemical etching at different times and substrate thickness on the optical properties of the material. The results express that the longer the time from 20 s to 180 s, the lower the reflectivity of the sample. Additionally, the thickness has little influence on the reflectivity, but the increase of thickness will increase the stability of the reflectivity. In 2019, K. Chen et al. [53] fabricated black silicon by MACE, and the researchers studied the effect of silver ion concentration on the optical and electrical performances of the material in the etching solution. The results show that the silver ion concentration has an effect on the porosity of black silicon samples, and the lower concentration of silver ion can improve the electrical performance, but has some damage to the optical performance.

In addition to the precious metals silver and gold, copper and nickel can be used in the catalytic metal nanoparticles of the MACE method. Due to the advantages of copper being cheaper and easily available, when compared with silver and gold metals, copper has a greater application prospect in the industrial application of black silicon production. Compared with wet chemical etching, black silicon fabricated by copper-assisted chemical etching has better optical absorption characteristics. B. Lee et al. [58] used Cu-assisted chemical etching to fabricate black silicon in 2020. First, Cu particles were deposited on the cleaned silicon wafer by CuCl_2_ and HF. Then, black silicon is fabricated in HF, H_2_O_2_, and H_2_O. The author optimized the Cu-ACE fabrication process of black silicon through a 100 W UV lamp. By comparing the black silicon prepared under different lighting environments, the black silicon prepared under the UV lamp environment has larger micropores and better light absorption. Ni-assisted chemical etching can also be used to fabricate black silicon materials. The researchers optimized the process steps by studying factors such as different etching time, different UV light illumination [59], and different etching solution ratios [60,61].

Compared to previous years, ultra-high aspect ratios and ultra-large pores have been fabricated on the surface of silicon in recent years. With recent development, the black silicon can achieve more special application through the unattainable process manufacture method. The black silicon fabricated by MACE has good performance in optical properties. In the fabrication process, the steps are simple, but the controllability of the surface morphology of black silicon is poor compared with the laser irradiation method. The cost of the MACE method is low, and it is expected to be used to fabricate black silicon on a mass scale to apply to black silicon solar cells.

### 2.3. Reactive Ion Etching

RIE [62] is a dry etching technique with strong anisotropy and high selectivity. The method uses an ion-induced chemical reaction to achieve anisotropic etching. That is to use the ionic energy to make the surface of the etched layer to form a damage layer that is easy to etch and to promote the chemical reaction. To fabricate black silicon with this method, the silicon wafer is first put into a vacuum chamber, and a corrosion gas is introduced to form a surface passivation layer. However, due to the instability of the passivation layer under the influence of plasma, the material surface is over-passivated, resulting in the black silicon structure. This etching method can damage the surface, pollute it, and it is difficult to form a finer structure. Equations (3)–(5) explain the chemical process of RIE method with SF6 and O_2_ as examples.

(3) $$Si+4F\left(g\right)\to SiF_{4}\left(g\right)$$

(4) $$SF_{6}\left(g\right)\to SF_{x}\left(g\right)+\left(6-x\right)F\left(g\right)$$

(5) $$SF_{x}\left(g\right)+Si+O_{2}\left(g\right)\to SiF_{y}\left(g\right)+SO_{z}$$

In 2013, M. Steglich et al. [63] fabricated black silicon by inductively coupled plasma reactive ion etching (ICP-RIE) in the pressure of 1 Pa or 4 Pa and a ratio of SF_6_: O_2_ in a gaseous environment of 1:1. Without the need for chemical pretreatment or (photolithography) masking of the chip, the optimal average reflectance of black silicon samples at the 300 nm to 1000 nm band is 5%. In 2014, M. Steglich et al. [64] used the ICP-RIE method to fabricate black silicon. The absorptivity was 99.5% at 350 nm to 2000 nm and 99.8% at 1000 nm to 1250 nm. When a layer of Al_2_O_3_ is formed on the black silicon by the ALD method, the absorptivity of the whole band can be improved. In 2015, H. Savin et al. [65] fabricated black silicon by deep reactive ion etching. Moreover, the surface is passivated by depositing 90 nm Al_2_O_3_ film, which further reduces the reflectance and the composition of surface carriers. The average reflectance of black silicon in the band of 300 nm to 1000 nm is around 3%. In 2016, M. Juntunen et al. [66] fabricated black silicon on 525 µm thick high-resistance n-type silicon wafers by ICP-RIE. The reflectivity of the black silicon material was gauged at different angles. The average reflectivity of the material at different angles from 350 nm to 1000 nm was below 10%. In 2018, K. Isakov et al. [67] fabricated broad-band absorption black silicon for niobium nitride deposition at the atomic layer using ICP-RIE technology. Black silicon was fabricated by etching the silicon surface with a mixture of SF_6_ and O_2_ gas at −110 °C. In the spectral region of 1100~2500 nm, the sample of niobium nitride layer coated with 15 nm achieved high absorption of 97%~99%.

**Figure 7:**
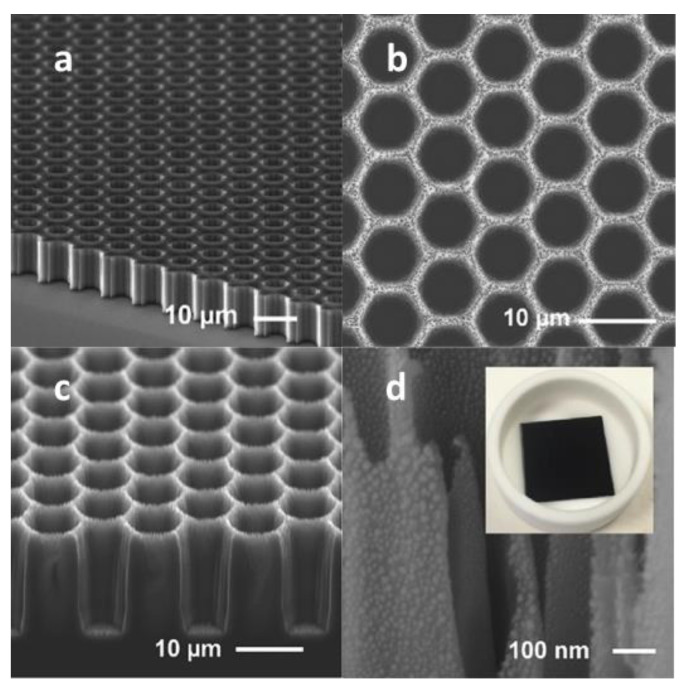
The SEM of order micro-nanostructure black silicon. (**a**) The cross-section SEM of black silicon after the first etching. (**b**) The top view of black silicon after the second etching. (**c**) The cross-section SEM of black silicon after the second etching. (**d**) The SEM of black silicon after Au deposition. Adapted from reference [68], with permission from Elsevier © 2020.

After several years of technological progress, regular surface morphology and smaller-scale micro-nano structures have gradually replaced single disordered surface structures. Due to the more repeated absorption of reflection, the multilayer micro-nano structures express more excellent performance in light absorption. Recently, multiply reactive ion etching is applied to fabricate excellent performance black silicon. In 2020, Z. Zhang et al. [68,69] combined two methods to fabricate order structure black silicon that has micro-nano structures on the surface of materials to apply for photothermal conversion. The silicon wafers were etched by reactive ion etching in the mask of photoresist to fabricate regular honeycomb-likely microstructure, firstly. Then, the microstructure material was etched by plasma etching in SF_6_ and O_2_ to fabricate nanostructure on the surface. Finally, the gold deposited on the order micro-nano structure black silicon to enhance the abruption of near-infrared waveband. The regular micro-nano structure black silicon has an ultralow reflectance of 1% and 5% in 200 nm to 1100 nm and 1100 nm to 1700 nm, respectively. Figure 7 shows SEM of order structure black silicon. The black silicon achieves excellent photothermal conversion compared with disordered nanopore silicon.

Compared with other methods, black silicon fabricated by the RIE method has an excellent broadband absorption rate. The black silicon fabricated by this method has a reflectance of less than 5% in both visible and near-infrared wavelengths, but it has some disadvantages in terms of cost due to the expensive equipment required.

### 2.4. Wet Etching

Wet etching is a technique in which the etched material is immersed in a corrosive solution. It is a pure chemical etching with excellent selectivity. The etch will stop when the current film has been etched without damaging the film of the next layer of other material. Because of the isotropy of all semiconductor wet etching, the width of transverse etching is close to the depth of vertical etching for both the oxide layer and the metal layer. Because of this isotropy, black silicon, fabricated in this way, forms tiny structures in the shape of micro pyramids on etched silicon wafers.

In 2017, Z. Qi et al. [70] fabricated black silicon material by a wet etching method. The surface of the pyramid-silicon was prepared by continuous etching for 30 min in a mixture of NaOH, NaSiO_3_, and isopropyl alcohol at 80 °C using a single side polished silicon wafer as the substrate. The average absorption rate of the black silicon at the wavelength of 1000 nm to 1600 nm is 55%. In 2018, P. Agnihotri et al. [71] fabricated black silicon materials by alkaline wet chemical etching. The researchers used KOH solution to chemically etch the polished p-shaped silicon wafers for 10 min at 80 °C. The wafer was washed with DI water and blow-dried to complete the fabricated conical microstructure with a uniform and high density on the surface. The aspect ratio of the conical microstructure is 1.3, and the angle between the sidewall and the base is about 55°. Black silicon materials have a 90% absorptivity from 300 nm to 2000 nm.

The black silicon fabricated by the wet etching method is larger in the microstructure. If there is no metal layer catalytic etching, it is usually pyramidal in SEM images. The depth of the microstructure is not as deep as that of black silicon made by other methods. Therefore, the absorption of black silicon in the region of visible light is not ideal without doping. However, it is cheaper and easier to fabricate black silicon by wet etching. This black silicon fabricated by wet chemical etching has been widely used in industrial production as the most advanced industrial technology. Through further optimization and exploration of this process, the performance of black silicon produced by this method can be further improved.

### 2.5. Plasma Immersion Ion Implantation Etching

PIII etching is a surface modification technique in which the accelerated ions in the plasma are injected into a suitable substrate by using a high-voltage pulsed DC or pure DC power supply as dopants. In the process of plasma immersion ion implantation, firstly the dissociated fluorine group will be injected into the silicon substrate and react with the silicon to form volatile SF4 gas, thus playing the role of etching. Then the product Si_x_O_y_F_z_ will deposit on the surface of the silicon to prevent the fluoro groups from continuing to react with the silicon, thus playing a part in passivation. Finally, the high-energy ions accelerated under the action of dc pulse bias will collide with Si_x_O_y_F_z_ deposited on the silicon surface, thus making the fluoro groups continue to react with the silicon. Under the joint action of the above three processes, the surface of the silicon wafer is formed into randomly distributed micro-spicules.

In 2011, Y. Xia et al. [21] fabricated black silicon by PIII, and the average reflectance of material at the wavelength from 300 nm to 1000 nm was 1.79%. In 2013, S. Zhong et al. fabricated black silicon with holes of different depths by PIII. Under different preparation situation, nanopores with a height of 150~600 nm were etched on the surface of the material. In the band from 350 nm to 1100 nm, the reflectance declined with the increment of the height of the nanopore, and the optimal average reflectance was 5%. In 2013, Y. Xia et al. [22] fabricated black silicon by PIII and researched the surface composition of the black silicon using XPS. The results show that the surface composition elements of the material are Si, C, O, and F, and the different surface structure was formed from porous to needle-like in the different gas ratio between SF_6_ and O_2_. When the microstructure of black silicon is porous and needle-like, the reflection of the black silicon is 4.87% and 2.12% at 200 nm and 1100 nm, respectively.

This method was applied to the dope element to expand the optical absorption range. In 2018, J. Lim et al. [72] combined plasma immersion ion implantation and plasma dry etching to fabricate high-performance black silicon. The substrates of P-type silicon were doped with N by the PIII process to form a PN junction, firstly. The PIII was in the N_2_ atmosphere. The second step of PDE was a sculptured nanostructure on the black silicon in H_2_ and Ar. The black silicon fabricated by the two steps method has an ultra-low reflectivity of 1.8% in 300 nm to 1100 nm.

Plasma immersion ion implantation is also a dry etching method, and more importantly, it is a method that can inject impurity elements into black silicon to expand the optical absorption range and promote optical absorption. This method can combine with other fabrication methods to fabricate order structure black silicon that has high absorption in the near-infrared band.

### 2.6. Electrochemical Method

This method has gradually developed since the electrochemical HF etching of silicon proposed decades ago. Electrochemical HF etching of silicon usually uses silicon as an anode and immerses it in a mixed solution of HF/H_2_O/ethanol. For the fabrication of black silicon, the current density, the proportion of HF, the etching time, and different light levels [73,74] are adjusted to control the morphology. According to the current density distinction, low current density and high current density will produce porous silicon and polished silicon, respectively, and the middle of these two current densities can produce random nanopillars without a mask. The exact current density of these three regions is determined by the doping type and concentration of the wafer. The n-type silicon wafer was electrochemically etched in a mixed solution of HF and MeOH to produce black silicon with low reflectance at 450 nm–1000 nm [75]. Since the black silicon produced by this method has different responses to light at different angles, the angle of incident light and the polarization-resolved reflectance coefficient of this black silicon were tested.

The above methods have their own advantages, but their methods involve toxic and corrosive chemicals that bring unknowing dangers. Recently, electrochemistry in molten salt is an emerging method of making black silicon. Another advantage of this method is that it does not involve expensive raw materials.

There are several different ways to prepare black silicon by molten salt electrochemistry. One of them is the production of black silicon by anodizing in molten salt [76]. By applying voltage between the two electrodes in the molten salt, through Si dissolution, redeposition, and Si alloying-dealloying with Ca to realize the production of black silicon. Another way [77] is to make black silicon by electro-reduction of SiO_2_ with microstructure.

The technical advantages of molten salt electrochemistry are that it is relatively simple and has no toxic or corrosive chemicals, and the price is also low. These advantages can provide feasibility for further expansion of production scale and industrial production of high-performance black silicon.

### 2.7. Comparison of Different Fabrication Methods

**Figure 8:**
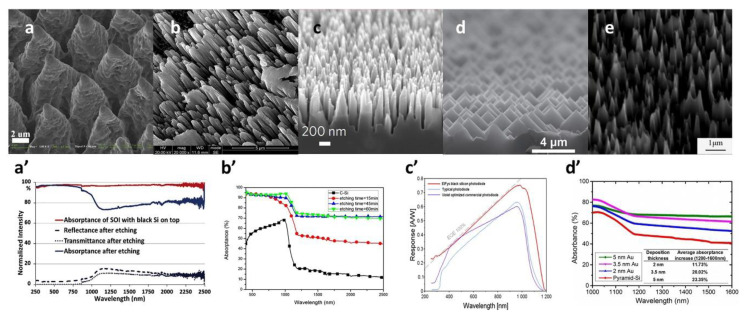
SEM and optical performances of using different fabrication methods. (**a**,**a’**) Black silicon using femtosecond/nanosecond laser irradiation. Adapted from reference [37], with permission from Elsevier © 2020. (**b**,**b’**) Black silicon using metal assistant chemical etching. Adapted from reference [50], with permission from Springer Nature © 2020. (**c**,**c’**) Black silicon using reactive ion etching. (**d**,**d’**) Black silicon using wet chemical. Adapted from reference [70]. (**e**) Black silicon using plasma immersion ion implantation etching. Adapted from reference [21], with permission from Elsevier © 2020.

Due to the different principles of fabrication methods, surface micro-nano structures with various morphologies are fabricated by diverse fabrication methods. Figure 8a,a’ show the SEM and optical performance of B-Si that is fabricated by femtosecond laser irradiation method. The optical performance clearly represents the advantages of the laser irradiation method that expand the optical absorption to 2500 nanometer wavelength by element doping. Fewer fabrication steps are another advantage of the laser irradiation method. However, the system of femtosecond laser irradiation method to fabricate black silicon is very expensive, which is a serious obstacle to mass fabrication of black silicon by this method. Figure 8b,b’ show the SEM and absorptance of B-Si fabricated by MACE. The MACE method has the advantage of a compromise between price and performance. This method can realize the control of the nanostructure on the black silicon through different metal nanoparticle deposition methods and different etching solutions. Figure 8c,c’ show the SEM and absorptance of B-Si fabricated by RIE. Compared with the absorption rate of polished silicon, the silicon etched by the RIE method has a very excellent improvement in the wavelength band below 1100 nm. Because there is an impurity energy level in B-Si, the absorption rate of black silicon also drops sharply. Figure 8d,d’ show the SEM and absorptance of B-Si fabricated by wet chemical etching. Wet chemical etching method is the low-cost fabrication method. The pyramid-like microstructure is one of the characteristics of this method. It is precisely because of this microstructure that the optical performance of this black silicon is much worse. Figure 8e shows the SEM and absorptance of B-Si fabricated by PIII. The reflectance is effectively suppressed in the wavelength of 300 nm to 1100 nm. However, more importantly, it is a method that can inject impurity elements into black silicon to expand the optical absorption range and promote optical absorption. The technical advantages of molten salt electrochemistry are that it is relatively simple and has no toxic or corrosive chemicals, and the price is also low.

## 3. Applications

### 3.1. Photocatalysis and Photo-Electrocatalysis

Since the discovery of the Honda-Fujishima Effect in 1967, photocatalysis has gradually become a hot topic in the field of catalysis. Photocatalysis has a very good application prospect in water purification, air purification, antifogging, degerming, and hydrogen and oxygen production. However, the existence of the electron recombination effect means the catalytic capacity is not high enough, and the efficiency of hydrogen production and oxygen production is not high enough. One of the effective methods is to weaken electron recombination by photo-electrocatalysis.

Black silicon, as one of the photo-electrocatalytic materials for hydrogen and oxygen production, has been used in recent years [78]. In 2011, the U.S. renewable energy laboratory [25] used black silicon as a cathode for photo-electrocatalytic hydrogen production. Due to the effective reaction area between the electrode and the solution increases, the cathode provides more positions for water-splitting and reduces the overpotential for photo-electrocatalytic hydrogen production. In 2020, S. Zhao et al. [79] fabricated serval black silicon using MACE and wet chemical etching to realize enhanced hydrogen generation. After the cleaning, the silicon was submerged in three different etching solutions to form a different surface morphology that has a different hydrophilic. The SiIP wafers act as a substrate to be electrodeposited metal Co, and the materials were sulfurated using CVD to finish the fabrication of photocathodes. The optimized photocathodes had a well onset potential of 0.22 V, and the photocurrent density is 10.4 mA cm^−2^ at −0.45 V. In 2020, B. Wang et al. [80] fabricated a plasmon-enhanced black silicon material to synthetic ammonia using photo-electrocatalysis. Through putting silicon into AgNO_3_ and HF solution, the Ag nanoparticles and surface nanostructure of black silicon were formed, due to the catalysis of Ag^+^ and the etching of HF. The fabricated Ag/b-Si photo-electrocatalytic had a superb alkaline air yield of 2.87 μmol h^−1^cm^−2^ at −0.2 V. There are three reasons for the high yield: The excellent light abruption of black silicon, the black silicon protection of Ag nanoparticles, and the co-catalysis of Ag. In 2019, B. Wang et al. [81] fabricated a material having highly efficient photo-electrocatalysis hydrogen evolution by modifying MoS_x_ quantum dot on the interface of material. The substrate of black silicon used the MACE method and MoS_x_ quantum dot used hydrothermal for 24 h in 240, was put into liquid nitrogen, and ultrasonicated for serval times to fabricate MoS_x_ quantum dot and black silicon synthesis by drop-casting. The material has an excellent photocurrent density of 12.2 mA cm^−2^, an onset potential of 0.255 V, and a H_2_ production rate of 226.5 μmol h^−1^ cm^−2^. In 2020, Y. Meng et al. [82] fabricated black silicon/(Ga_1−x_Zn_x_) (N_1−x_O_x_) nanorods to use as photo-electrocatalysis water splitting. Researchers used the MACE method, water bath, heating after dripping, and nitridation to fabricate the photo-electrocatalysis material. Due to the better absorption of black silicon, the grown (Ga_1−x_Zn_x_) (N_1−x_O_x_) nanorods on surface structure had better performance compared with growing on polished silicon. The result shows that black silicon/(Ga_1−x_Zn_x_) (N_1−x_O_x_) nanorods have 0.55 μA cm^−2^, approximately 5 times more than (Ga_1−x_Znx) (N_1−x_O_x_) nanorods/polished silicon.

**Figure 9:**
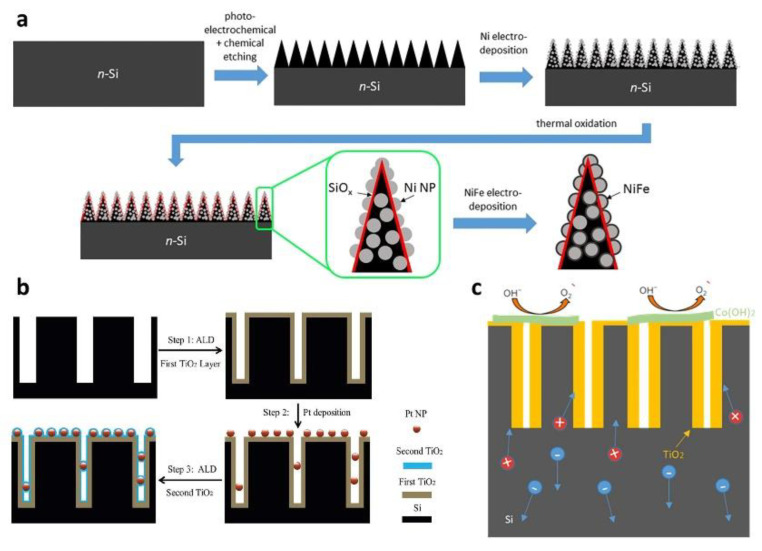
The applications of black silicon in photocatalysis and photo-electrocatalysis. (**a**) The fabrication process of NiNPs and SiO_x_ on the black silicon surface. (**b**) The fabrication process of double TiO_2_ protective layers with Pt nanoparticles on black silicon. Adapted from reference [26], with permission from Royal Society of Chemistry © 2020. (**c**) Schematic illustration of the charge generation and oxygen evolution processes in b-Si/TiO_2_/Co (OH)_2_.

Since the stability of the catalyst cannot be guaranteed in the long-time reaction, researchers began to explore the protection of the black silicon photo-electro-catalyst. In 2016, the U.S. renewable energy laboratory [26] used MACE to make black silicon, and platinum nanoparticles were chemically deposited to fabricate photocathode. Figure 9b shows the framework of black silicon photo-electrocatalysis. It was found that PtNPs could enter the silicon by secondary metal-assisted etching. Moreover, due to the constancy of the Pt/Si interface, platinum nanoparticles’ black silicon photocathode has a stable hydrogen evolution activity. In 2017, the University of Wisconsin–Madison used the atomic layer deposition (ALD) method to deposit titanium dioxide protective film and cobalt hydroxide film on black silicon as the photo-anode of photo-electrocatalysis [83]. Figure 9c shows the charge generation and oxygen evolution processes in photo-electrocatalysis. The nano-heterostructure has a photocurrent density of 32.3 mA cm^−2^ at an applied voltage of 1.48 v with cobalt hydroxide film under the sunlight. The titanium dioxide film passivated the defective sites on the interface without affecting the light absorption and charge transfer characteristics to improve charge separation efficiency and photocurrent density of photo-anode. Moreover, the titanium dioxide film isolates black silicon from the alkaline electrolyte, improves the catalytic stability of the black silicon, and prolongs the service time of the black silicon structure. In 2017, black silicon was fabricated by deep-reaction ion etching (DRIE) [84]. Water splitting photocatalyst was prepared by the deposition of 40 nm titanium dioxide and sputtering 40 nm gold film on the surface of the material. The wide wavelength absorption of substrate helps titanium dioxide films to have better photocatalytic capability. However, the intrinsic surface morphology of black silicon hinders the hydrophilicity of photocatalytic films. In 2019, San Diego State University fabricated black silicon from metal-assisted etching silicon wafers [85]. The researchers sequentially deposited titanium dioxide films, platinum nanoparticles, and titanium dioxide films on the surface to achieve the protection of strong acids and strong bases. In 2019, researchers [86] fabricated the black silicon by combining with photochemical and chemical etching methods. After the formation of NiNPs and SiO_x_ on the material surface, NiFe was deposited to make a photoanode without a protective layer. Figure 9a shows the fabrication process of the photo-electrocatalysis material. The photoanode can produce oxygen at a steady high rate for 16 h.

Photo-electrocatalysis usually has a low performance due to poor light absorption capacity. The excellent light absorption ability of black silicon can promote the performance of photo-electrocatalysis when it is used as a substrate of active material. Combined with black silicon and active material, the active material can have better efficiency due to the multiple reflections in the nanostructure of black silicon. Due to the durability of photoelectric catalysis, the micro-nano structure on black silicon can very easily cause damage after a period of photoelectric catalysis, which reduces the photoelectric catalytic ability. Therefore, researchers have used different methods to protect the surface structure of black silicon, aiming to extend the stability of photo-electrocatalysis.

### 3.2. Near-Infrared and Visible Light Photodetection

Near-infrared band photodetection has many applications in engineering, medical treatment, and detection. For example, near-infrared detectors can be used for non-contact temperature measurement, gas composition analysis, nondestructive flaw detection, thermal image detection, infrared remote sensing, and military target detection, search, tracking, and communication. Along with the evolution of modern technology, the application prospect of the infrared sensor will be broader. Therefore, it is very important to increase the absorption rate and absorption range of NIR detection. Black silicon has excellent absorption and low reflectance in the NIR band. Since the late 1990s, when Eric Mazur, a professor at Harvard University, discovered black silicon by accident [9], researchers have been exploring its application to infrared photodetection [47,63,82,83,84,85,86,87,88,89] and ultrahigh sensitivity in the ultraviolet band and visible light range [90,91,92].

**Figure 10:**
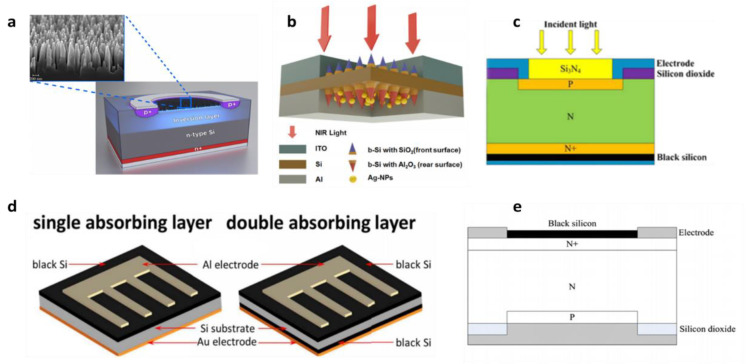
Photodetector based on black silicon with different principles and structures. (**a**) Cross-section of induced-junction b-Si photodiode structure with high EQE in the 250 nm to 950 nm. (**b**) Schematic of b-Si/Ag-NPs Schottky photodetector. Adapted from reference [52]. (**c**) Structure of Si-PIN photoelectronic detector with microstructure black silicon at the back surface. Adapted from reference [50], with permission from Springer Nature © 2020. (**d**) Two different schematic diagrams of nitrogen-doped black silicon photodiodes with single layer or double layers black silicon. Adapted from reference [93], with permission from IEEE © 2020. (**e**) Structure of Si-PIN detector based on nanostructured black silicon. Adapted from reference [94].

Due to its low reflectivity, black silicon can be used as the surface of the absorption layer to absorb near-infrared light, to enhance the photodiode absorption of light. In 2016, M. Juntunen et al. [66] of Aalto University in Finland formed a PN-like structure by injecting boron ions and phosphorus ions into the positive/negative side of black silicon. Figure 10a shows cross-section of photodetection. Then, the researchers created photodiodes by depositing Al layers in the front/back by atomic layer deposition. The external quantum efficiency (EQE) is as high as 96% in the 250 nm to 950 nm band, and the spectral responsivity is close to the ideal photodiode.

When black silicon fabricated by different fabrication methods is applied to photodetectors, due to the difference of surface nanostructures, the responsivity and response range will have certain differences in the near-infrared band. In 2016, H. Zhong [50] fabricated B-Si materials by MACE. The light absorption rate of black silicon is greatly enhanced, and the maximum absorption rate can reach 95% in the wide wavelength range of 400~2500 nm. Figure 10c displays the framework of Si-pin photodetection. The Si-pin photodetector based on the material has a response rate of 0.57 A/W at the 1060 nm. In 2018, H. Zhong et al. [94] fabricated black silicon using a two-step method combining DRIE and PIII. Figure 10e shows the structure of Si-pin photodetector. Si-pin photodetector using this black silicon material is 0.31 A /W and 0.53 A /W, respectively, at 1060 nm and 1100 nm. Although the second method reduces the response rate at the same wavelength, the surface nanostructures are more regular, broadening the detector’s response range.

The researchers further improved the light response range of this special material detector in the near-infrared band by utilizing the surface plasmon resonance effect of the metal nanoparticles [95]. In 2017, Z. Qi et al. [70] fabricated a gold nanoparticle modified silicon pyramid-shaped material that is able to enhance thermal electron NIR light detection. The thermionic detector using this material has a wide light response spectrum in 1200~1475 nm, and the dark current is 10^−5^ Acm^−2^. In 2019, F. Hu et al. [52] designed a sub-band-gap black silicon Schottky photodetector with AgNPs deposited on the back. Figure 10b shows the schematic of b-Si/Ag-NPs photodetector. At the reverse bias voltage of 3 V, the response rates of the b-Si/Ag-NPs photodetector in 1319 nm and 1550 nm were 0.277 mA/W and 0.226 mA/W, respectively. Table 1 shows the performance comparison of photodetectors based on black silicon in the past ten years.

The thermal annealing process is a significant element that influences the light absorption rate of black silicon and the light response rate of the detector. It is also one of the research directions to study the effect of reducing the step of hot annealing on the preparation of black silicon materials. In 2018, C. Li et al. [93] fabricated a black silicon Schottky photodiode by plating Al and Au metal electrodes on the front and back of the silicon. Figure 10d show different structure Schottky photodetection. The broadband photodiode has good thermal stability, and the optical response rate at the 1330 nm band is 5.3 mA/W at 10 V reverse bias. In 2020, S. Huang et al. [88] promoted the performance of black silicon photodetection by using rapid thermal annealing and surface passivation. The a-Si: H layer is deposited on the surface of material using a plasma-enhanced chemical vapor deposition (PECVD) method to reduce dark current, and the rapid thermal annealing can diminish the diffusion of sulfur doping to lower the degree of the defect. The black silicon photodetection acquired an excellent performance, where the response rate is 0.80 A/W at 1550 nm and the dark current is 7.8 μA at −5 V.

The photodetector based on black silicon material has a high spectral responsivity and a wider spectral response range, especially because the doped black silicon material has a much higher responsivity than silicon materials in the near-infrared band. Firstly, researchers use black silicon as the light absorption layer of the photodetector and increase the responsivity of the photodetector through the ultra-low reflectivity of black silicon. The photodetectors made by different black silicon fabrication methods usually have different response ranges and responsivity due to the different surface morphology and doping. Currently, the higher absorption of material brings the possibility of higher responsivity to black silicon-based photodetectors. Secondly, since undoped black silicon has low absorption in the NIR band, the surface plasmon resonance effect formed by noble metal nanoparticles is used to promote the absorption of undoped black silicon in the NIR band. Thirdly, the effect of annealing on black silicon is also one of the most significant research directions.

### 3.3. Solar Cell

The use of fossil fuels has brought about vigorous economic development to society and brought a lot of convenience to people. However, fossil energy is also limited, its combustion causes environmental pollution, and the greenhouse effect also affects the earth’s ecology and climate. As people began to realize that relying on fossil fuels alone was not feasible, researchers began to explore environmentally friendly, renewable energy sources. Solar energy can be seen everywhere on the earth, and the utilization of solar energy as new energy has become one of the research directions of researchers. The application of solar cells has already entered the fields of industry, commerce, agriculture, communication, household appliances, and public facilities from the military and aerospace fields. In particular, it can be distributed in remote areas, mountains, deserts, islands, and rural areas to save on costly transmission lines. In recent years, black silicon has become one of the materials used to make solar cells [72,97,98,99,100,101]. The research hotspot is to change the surface structure of black silicon [102] and passivate black silicon [103].

**Figure 11:**
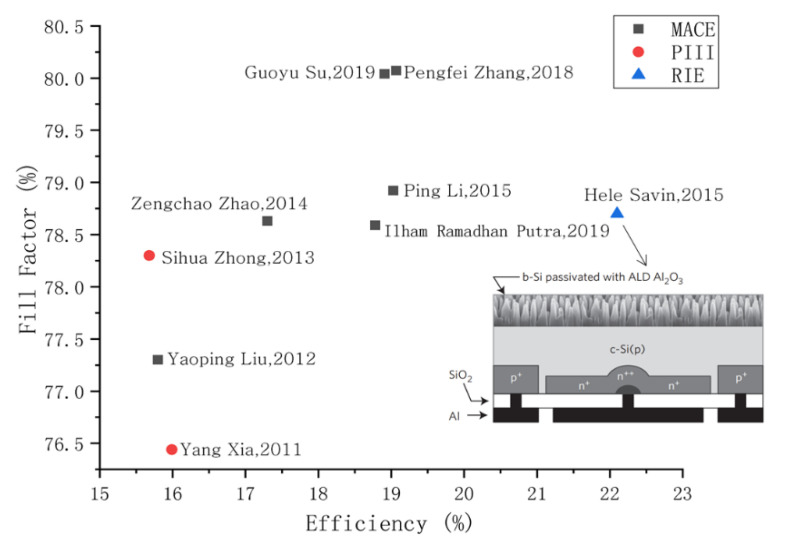
Performances comparison of solar cells based on black silicon, the inset shows black silicon solar cells with interdigitated back-contacts.

By changing the surface structure of the material in different ways, the researchers can improve the conversion efficiency of B-Si solar cells. Figure 11 shows the performance comparison of solar cells based on black silicon. In 2014, Z. Zhao et al. [49] fabricated black silicon by MACE. The surface treatment of B-Si by tetramethyl-ammonium hydroxide has better internal quantum efficiency than that of untreated black silicon in the short-wave region. The surface of black silicon was etched with tetramethyl-ammonium hydroxide, and the microstructure of black silicon changed slightly, resulting in a slight increase in reflectivity. The tetramethyl ammonium hydroxide (TMAH) etching treatment also changed the doping concentration of black silicon, and the depth of heavy doping concentration became shallower. This may inhibit carrier auger recombination of the b2-base cell diffusion emitter. The filling factor of the black silicon solar cell was 78.63%, and the conversion efficiency was 17.3%. In 2016, P. Li et al. [99] treated the black silicon surface with tetramethylammonium hydroxide for the 30 S, and obtained a black silicon solar cell with a better conversion efficiency, with a filling factor of 78.80% and a conversion efficiency of 19.03%. In 2018, G. Su et al. [104] fabricated black silicon material using metal-assisted chemical etching of diamond wire saw polysilicon. The researchers removed the etching process at different times to form nanostructures of different sizes on the surface. The defect removal etching (DRE) process can affect the nanometer size and reflectance of material, etc. With the increase of DRE time, the reflectance becomes larger and the passivation effect becomes better. The DWS MC-Si solar cell filling factor was 80.07%, and the conversion efficiency was 19.07%. In 2019, I. Putra et al. [51] made a B-Si solar cell by performing a silver-assisted chemical etching of the micro pyramid on a silicon wafer to form a finer nanocolumn structure on the micro pyramid. The increase in the formation time of Si nanopores led to a significant increase in photoluminescence properties. This may be because charge composition limits the efficient separation of photogenic carriers. The sample filling factor with an etching time of 8 min was 78.59%, and the conversion efficiency was 18.78%.

For solar cell, the recombination of photogenerated electrons and holes on the material surface is a major factor that limits the efficiency of solar cells. Due to the further in-depth research, how to avoid the recombination of photogenerated electrons and holes has gradually become one of the research hotspots. By surface passivation of B-Si, it reduces the surface photocarrier recombination rate and improves the conversion efficiency of B-Si solar cell. In 2015, H. Savin et al. [65] fabricated black silicon by deep reactive ion etching. Moreover, the surface was passivated by depositing 90 nm Al_2_O_3_ film, which further reduced the reflectance and the composition of surface carriers. The researchers chose an interdigitated back-contact back-junction (IBC) type of solar cell structure, which relies heavily on the surface composition of smaller photocarriers to improve efficiency. The Figure 10 inset illustrates the structure of a solar cell. The filling factor of the B-Si solar cells obtained by the method is 78.7%, and the conversion efficiency is 22.1%. In 2019, D. Kim et al. [105] treated black silicon materials with Al_2_O_3_ films by using (NH_4_)_2_S solution. It slows down the recombination of the surface electrons. The researchers soaked black silicon with 10 nmAl_2_O_3_ films in (NH_4_)_2_S solution at different times. The results showed that the sample immersed in the solution for 10 min had better conversion efficiency. The conversion efficiency reached 13.5%, the filling factor was 72.02%, and the sample conversion efficiency increased by 16% before the comparison treatment.

The surface structure of the solar cell light-absorbing material is one of the key factors to promote the performance of the solar cell. As the most widely used solar cell material with the largest market share, silicon material has a mature industrial chain in terms of processing. As a material with ultra-low reflectivity and ultra-high absorptivity in the visible light and NIR bands, black silicon is applied to solar cells, greatly improving their efficiency. Researchers have done a lot of research on the surface structure of black silicon and the surface passivation of B-Si solar cells, but further exploration is needed in terms of cost reduction and mass production.

### 3.4. SERS

Surface-enhanced Raman scattering (SERS) is a technique used to detect and analyze compounds. Because of its ultra-high sensitivity, resistance to photobleaching, narrow spectral bandwidth, and molecular feature detection, SERS is applied in many spheres such as food safety, biological sciences, environmental monitoring, and other fields. In general, the substrate used for SERS detection is a rough surface composed of noble metals. In recent years, due to the large surface area and/or high aspect ratio of black silicon structures, it has attracted widespread attention as the potential SERS substrate.

**Figure 12:**
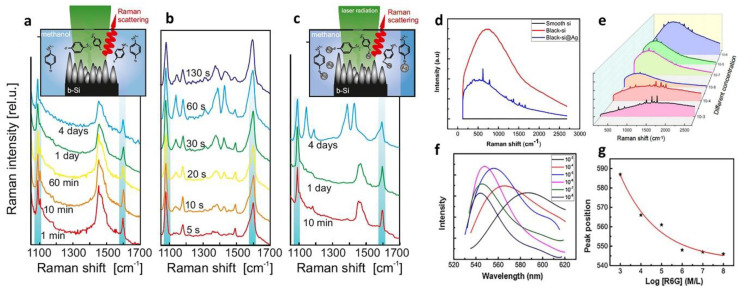
Application of black silicon in surface-enhanced Raman scattering (SERS). (**a**–**c**) detected PATP-to-DMAB photocatalysis conversion using different bare or Ag-coated black silicon with or without laser radiation in the aqueous methanol solution. Adapted from reference [103]. (**a**) Bare black silicon without laser radiation. (**b**) Ag-coated black silicon without laser radiation. (**c**) Bare black silicon without laser radiation. (**d**–**g**) Performances of R6G concentration detector based on black silicon. Adapted from reference [107], with permission from Elsevier © 2020. (**d**) Sensitivity of black-silicon SERS substrate. (**e**) Original SERS spectrum of different R6G concentration. (**f**) Fluorescence spectra of different concentrations of R6G. (**g**) The corresponding R6G sensor calibration diagram drawn by different fluorescence peaks.

In 2018, E. Mitsui [106] proposed a black silicon structure that is chemically inert and consists of randomly-arranged pointed Mie resonators, used to identify low-concentration the molecular fingerprints without contact. By comparing the SERS signal of the catalytic conversion of para-aminothiophenol (PATP) to 4,4‘-dimercaptoazobenzene (DMAB) between metal-coated black silicon and metal-free black silicon, it demonstrated the non-contact ultra-sensitive SERS detection of metal-free modified black silicon at concentrations as low as 10^−6^ M. Figure 12a–c show the different situation substrates detected in PATP-to-DMAB photocatalysis conversion.

In 2019, L. Liu [107] fabricated a black silicon substrate with high sensitivity of the SERS signal by using the plasma immersion ion implantation method. Testing and calibration of different concentrations of R6G show a good relationship between the fluorescence peak and the concentration, and the calibration plot coefficient is as high as 97.8%. Figure 12d-g shows the relationship between fluorescence peak and concentration. Therefore, the concentration of R6G can be detected by using a black silicon substrate to detect the change in fluorescence peak. When the concentration of R6G changed from 10^−8^ M to 10^−3^ M, the fluorescence peak shifted from 545 nm to 588 nm. The fluorescence peak shows a red-shift phenomenon with increasing concentration, which may be caused by the repulsive force field of other molecules.

Surface-enhanced Raman vanquishes the shortage of low Raman spectroscopy sensitivity and can obtain structural information that is not easily obtained by conventional Raman spectroscopy. It is widely used in surface research, adsorption interface surface state research, interface orientation, and configuration of biological large and small molecules, conformation studies, structural analysis, etc., can analyze the adsorption orientation of the compound at the interface, the change of adsorption state, and interface information. The use of black silicon as the substrate greatly enhances the SERS signal and increases the detection sensitivity.

### 3.5. Sensing

The application of black silicon in sensing is not as popular as solar cells and photodetectors. However, the sensitivity of sensors based on black silicon is usually very high, that is to say, sensors based on black silicon materials can detect very weak signals. Because of this unique advantage, black silicon sensors have gradually developed in recent years. Black silicon sensors are mainly used in gas sensing and imaging. In gas sensing, it is usually used in the detection of ultra-low concentration gases, such as the detection of dangerous gases. In an imaging sense, there are already products for imaging under low light conditions.

**Figure 13:**
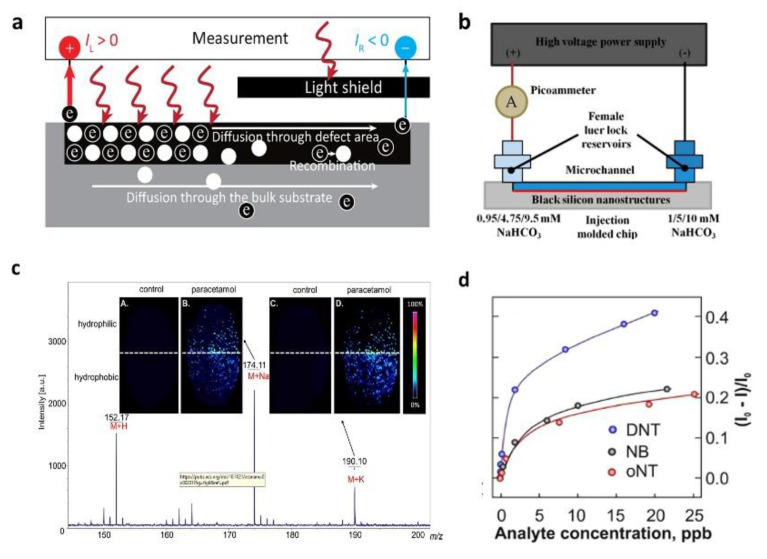
Serval applications based on black silicon in sensing. (**a**) Structure of ammonia sensor using b-Si material to realize ultrahigh Sensitivity. Adapted from reference [108], with permission from American Chemical Society © 2020. (**b**) Experimental setup for black silicon nanostructures current monitoring. Adapted from reference [109], with permission from Royal Society of Chemistry © 2020. (**c**) Using black silicon to achieve surface-assisted laser desorption/ionization mass spectrometry imaging. Adapted from reference [110], with permission from American Chemical Society © 2020. (**d**) Evaluate detection limits of cbz-bSi gas sensor. Adapted from reference [111], with permission from American Chemical Society © 2020.

In 2018, X. Liu [108] first proposed the use of black silicon materials as gas sensing materials. Based on the excellent photoelectric characteristics of the black silicon material, the researchers realized the dual drive sensor of light and electricity by asymmetrically illuminating the two electrode regions. Figure 13a shows the structure of the ammonia sensor using b-Si material. Within a certain range of applied voltage, the sensitivity is significantly improved and tends to infinity. Foraging equipment with reduced sensitivity, the sensitivity to 500 ppm NH_3_ will increase by two orders of magnitude with an additional optical drive. Finally, a mechanism based on the Dember effect explained this phenomenon.

In 2018, A. Lim et al. [109] studied the effect of nanostructured black silicon on the electroosmotic flow effect, which was used to accurately control the fluid velocity in microfluidic chips. The researchers have proposed a new technique that uses dry etching, electroplating, and molding (DEEMO) processes and RIE to fabricate long-silicon nano-silicon microchannels. The reduction of EOF is caused by the local electric field distortion on the nanostructure, and the longer the B-Si nanostructure, the better the effect of reducing the velocity. Figure 13b shows the experimental setup for black silicon nanostructures’ current monitoring. The experimental results show that the EOF speed is reduced by 13.7%, which is quite close to the simulation result predicted to reduce by about 8%.

In 2019, A. Mironenko [111] fabricated a black silicon-based ultra-sensitive detector by attaching a carbazole monolayer on the surface of nanostructured black silicon. It can achieve a sensitivity test as low as 10^−12^ and a dynamic test range as high as 10 PPM. Figure 13d shows the comparison of FL quenching efficiency in different targets. Due to the presence of the carbazole monolayer, the luminescence spectrum of the reaction between the carbazole monolayer and the aromatic nitro molecule can be used to identify the presence of the aromatic nitro molecule. While the sensor has high sensitivity, it also has an ideal dynamic test range. This is precisely because the nanostructure on the surface of the B-Si substrate makes the local concentration of carbazole molecules uneven, forming surface reaction sites with different sensitivities. The sensor can be used to detect dangerous components in air or water to prevent pollution and danger.

In 2020, S. Iakab [110] fabricated a black silicon substrate modified with gold nanoparticles for surface-assisted laser desorption/ionization mass spectrometry imaging. Figure 13c shows the performance of black silicon used as SALDI-MSI. To this end, the researchers used a variety of plasma treatment methods to modify the surface of gold-modified black silicon to form hydrophilic and hydrophobic gold-modified black silicon, which can selectively adhere to molecules.

### 3.6. Antibacterial Material

One of the main challenges facing the biomedical industry is the development of powerful antibacterial surfaces. Due to the needle-like nanostructure of black silicon, black silicon has also been used as one of the antibacterial materials in recent years.

**Figure 14:**
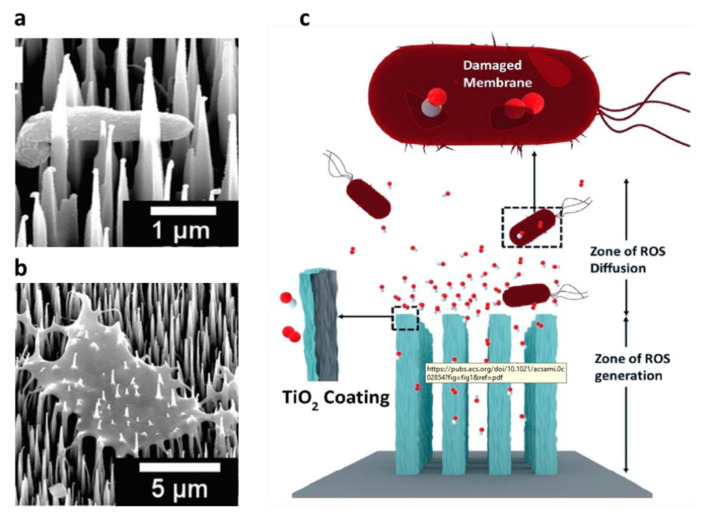
Applications of black silicon as antibacterial material. (**a**) SEM of bacteria trapped in black silicon. Adapted from reference [112], with permission from Royal Society of Chemistry © 2020. (**b**) SEM of bacterium punctured by nanostructures on black silicon surface. Adapted from reference [113]. (**c**) Reactive oxygen species (ROS) is produced by the photocatalysis of TiO_2_ to decompose bacteria. Adapted from reference [114].

The researchers conducted antibacterial studies on black silicon with different nanostructures, such as the height of needle-like structures and surface density. In 2018, G. Hazell et al. [112] used the RIE method to fabricate needle-shaped black silicon surfaces with different depths and surface densities for use as antibacterial materials. The researchers studied the killing effect of black silicon and black diamond surfaces on Gram-negative (e. Coli) and Gram-positive (s. codon) bacteria. All black silicon with nanostructures killed E. coli significantly faster than planar silicon or diamond samples. Compared with the density of the needle-shaped surface of black silicon, the length of the needle-shaped surface is a more important factor for the sterilization effect. Figure 14a,b show that the bacteria sink or are impaled by the nanostructure of black silicon. In contrast, Gordonii is not affected by the surface of the nanostructure. Because of their smaller size, thicker cell membranes, and lack of mobility. In 2018, C. Bhadra et al. [113] studied the effect of B-Si nano-surface structures with different heights and surface densities on antibacterial effects. The results of this work prove that although B-Si substrates have visually similar nanostructures, the sterilization efficiency of these substrates is different. For different bacteria, different structures of black silicon have different bactericidal effects, and the specific laws need further research.

In 2020, J. Singh et al. [114] studied the antibacterial ability of black silicon and black silicon oxide deposited with a photocatalytic layer of titanium dioxide. This antibacterial material combines the good light absorption capability of black silicon and black silicon oxide with the antibacterial mechanism of photocatalytic materials. Figure 14c shows the principle of antibacterial using black silicon or black silica. Photocatalytic materials generate active oxygen by decomposing surface water molecules. Due to the strong oxidative decomposition ability of this substance, the bacterial cell membrane is decomposed and the antibacterial ability is improved. Although high nanostructures absorb more light, finite-difference time-domain (FDTD), and finite element simulation results show that the higher the nanostructures are, the higher the ROS concentration is not a certainty. It may be because the nanostructure exceeds 5 microns in height, the ROS cannot diffuse out of the nanostructure.

The B-Si with a needle-like structure on the surface can puncture bacteria with a sharp tip, destroying the bacterial structure and inactivating it. However, smaller-scale bacteria are not easily destroyed by the needle-like structure. By combining the antibacterial ability of the photocatalytic material with the needle-like structure of black silicon, the better antibacterial ability can be achieved.

## 4. Summary and Future Directions

Along with the quick progress of the semiconductor industry, silicon is becoming one of the most popular semiconductor materials, due to its small size, wide range applications, easy integration, and long life. However, silicon materials have some disadvantages, such as high reflectivity and wide-bandgap. The black silicon remedies these disadvantages to realize low reflectivity at 350 nm to 2500 nm and excellent optical and electrical properties. After the discovery of black silicon, researchers began to study the application of black silicon in the photodetector. At the same time, they began to study the different methods to fabricate black silicon and the factors affecting the properties of black silicon materials. After more than 20 years of development, the fabrication process of black silicon materials has gradually developed through several different technologies, such as femtosecond laser pulse irradiation, RIE, MACE, wet chemical etching, and plasma immersion ion implantation etching. In recent years, with the development of the fabrication technology of black silicon materials, the application of black silicon materials has also been explored by researchers. Black silicon material has many applications in the fields of photoelectric detection, photothermal conversion, solar cell, photoelectric catalysis, and sensing.

In terms of the fabrication of black silicon material, it will proceed towards mass production and order surface morphology. Instead of simply etching the silicon wafer surface, it will design the surface micro-nano structure and produce high-performance black silicon through accurate fabrication methods. For example, photolithography is combined with plasma injection reactive ion etching or Bosch etching with deep reactive ion etching to fabricate order micron structures and nanostructures on micron structures. There are a lot of literatures on the optical properties of black silicon. In terms of electrical characteristics research, there is a lack of systematic and unified research. In some applications of black silicon, such as photocatalytic applications and photodetection applications, the research on the photoelectric properties of black silicon is involved, but there is still a lack of special research on the electrical properties of the material. We believe that the research on the electrical characteristics of black silicon also needs further exploration in the future.

There will also be more and more efficient applications. In the field of the photodetector, because the black silicon with micro-nano structure breaks through the inherent optical absorption wavelength limit of silicon materials, it will have high absorption in the near-infrared band. Therefore, achieving high responsiveness in optical communication bands such as 1330 nm and 1550 nm will be one of the development trends of photodetectors using black silicon. Due to the mature micro-nano processing technology of silicon material and highly integrated, the great absorption of black silicon can fabricate high highly responsive photodetector in visible light to near-infrared to use as wide spectrum detection. In solar cells, because silicon is cheaper than other solar cell materials, black silicon has a large market in the future as a solar cell material. Research is advancing towards the mass production of large black silicon solar cells. In polycrystalline solar cells, black silicon material also has great advantages. So, it has great development and application potential in polycrystalline black silicon solar cells. In catalysis, black silicon will combine the high-absorption structure of near-infrared and visible light with the high-efficiency photoelectric catalytic material to produce hydrogen and oxygen efficiently and synthesize ammonia with nitrogen. Besides, researches will be carried out on the long-lasting efficacy of catalysis. There will be more breakthroughs and applications in other directions, such as photoluminescence, antibacterial materials, and high-sensitivity sensing.

## Figures and Tables

**Table 1:** Performance comparison of photodetectors based on black silicon.

Year	Nation	Mechanism	Main Response Band	Performance	Ref.
2013	China	MSM-bSi	600 nm	76.8 A/W	[47]
2013	Germany	PtSi-bSi	1550 nm	EQE: 27%, 0.33 A/W	[65]
2014	China	AuNPs@graphere/CH3- SiNWs	850 nm	1.5 A/W	[96]
2014	China	MSM-bSi	-	0.5 A/W@5 V	[48]
2016	China	Si-Pin	1060 nm;1100 nm	0.57 A/W; 0.37 A/W	[50]
2016	Finland	the induced-junction PD	250 nm–950 nm	EQE: 96%	[66]
2017	China	AuNPs-bSi	1200 nm–1475 nm	8.17 mA/W	[79]
2018	China	Schottky-based	1310 nm	5.3 mA/W@10 V	[93]
2018	China	Si-Pin	1060 nm	0.53 A/W	[94]
2019	China	bSi/AgVPs PD	1319 nm; 1550 nm	0.277 mA/W; 0.226 mA/W@3 V	[52]
2020	China	Schottky-based	650 nm; 808 nm; 1550 nm	445 mA/W; 56 mA/W; 15 mA/W@8 V	[89]

## Data Availability

The data presented in this study are available on request from the corresponding author.
